# Visualization of grout diffusion in coarse-grained materials using transparent soil: Effects of Fine particle content

**DOI:** 10.1371/journal.pone.0350629

**Published:** 2026-06-01

**Authors:** Shihao Zhang, Jianxin Wang, Zhuo Li, Yusheng Li

**Affiliations:** 1 College of Hydraulic and Civil Engineering, Xinjiang Agricultural University, Urumqi, Xinjiang Uygur Autonomous Region, China‌‌; 2 Xinjiang Agricultural University, Xinjiang Key Laboratory of Hydraulic Engineering Security and Water Disasters Prevention, Urumqi, Xinjiang Uygur Autonomous Region, China; Instituto Federal do Espírito Santo: Instituto Federal de Educacao Ciencia e Tecnologia do Espirito Santo, BRAZIL

## Abstract

Fine-particle loss in earth-rock dams can induce abnormal grout diffusion during rehabilitation. To address this issue, we investigated the influence of fine particle content on grouting efficiency in coarse-grained materials. Using transparent soil technology, coarse-grained materials were simulated with fused quartz sand and a refractive-index-matched pore fluid (n = 1.4585). Dyed epoxy resin was used as a cement grout analog. Constant-pressure grouting tests (40 kPa) were performed on three test conditions representing no fine-particle loss, partial fine-pareicle loss, and complete fine-particle loss. The grout diffusion process was visualized and quantified using Particle Image Velocimetry (PIV). The results reveal that fine particle content critically controls grout diffusion patterns and rates. (1) Excessive fines cause pore clogging, resulting in grout upwelling, surface seepage, and limited diffusion, forming locally consolidated masses with blocky bonding. (2) An appropriate fine particle content enables uniform spherical diffusion, creating an optimized structure characterized by point bonding of large particles and small-pore filling. (3) The absence of fines leads to gravity-dominated rapid settlement with weak horizontal diffusion, leaving only surface-coated particles. This study elucidates the coupled mechanisms of fine-particle migration, clogging, and grout diffusion, providing an experimental basis for optimizing permeation grouting in coarse aggregates.

## Introduction

Earth-rock dams are susceptible to seepage and instability due to factors such as earthquakes and differential settlement. These defects often involve the loss of fine particles from the dam shell material. This may lead to accidents. Therefore, grouting methods are employed to reinforce coarse-grained materials, thereby enhancing the stability and durability of earth-rock dams. Grouting, as a specialized construction technique, possesses short construction cycles, rapid reinforcement effects, and relatively minimal environmental impact compared to other treatment methods. Consequently, this technique has been extensively applied in tunnel reinforcement, roadway stabilization, and foundation treatment. In actual projects, however, the grouting effect varies depending on the fine particle content in the injected area, which significantly influences whether the reinforcement achieves the desired outcome. High fine-particle content can prevent grout from effectively dispersing within the injected medium, whereas low fine-particle content can induce it to spread too rapidly, preventing proper bonding with the medium. In both cases, re-grouting may be required.

Numerous scholars worldwide have conducted research on grouting. Some researchers [1] performed grouting tests in rock fractures and observed increasing difficulty with prolonged injection duration. Han et al. [2], conducting tests in gravelly sand and sandy soil layers, demonstrated a sharp decline in grout velocity with increasing radial distance. However, traditional grouting tests struggle to observe the in situ diffusion process of grout in real time, typically relying on post-grouting excavation to analyze the consolidated grout body morphology. Consequently, this post-grouting excavation method cannot reveal the dynamic diffusion mechanisms, as it only allows for assessment after the grout has solidified [3]. Transparent soil technology enables effective observation of soil changes during laboratory experiments. Simulated grouting tests using transparent soil have revealed spherical or ellipsoidal diffusion patterns consistent with Maag’s spherical diffusion theory. Current grouting research primarily focuses on sandy soils and rock fractures, with limited studies on coarse-grained materials. While most analyses emphasize factors like grouting pressure [4], rate [5], and temperature [6], the fine-particle content within the grouted zone also significantly impacts effectiveness. Furthermore, using actual sandy soils and opaque similar materials makes it difficult to effectively observe grout flow and diffusion during the grouting process. Transparent soil technology is primarily applied in pile foundation [7,8] and tunnel engineering [9,10], addressing soil displacement [11], deformation [12], and seepage [13] issues. Research on grout flow direction [14] within transparent soils remains scarce, with existing studies mainly confined to well-graded gravel overlays with maximum particle sizes of 5 mm [15]. The diffusion behavior of grout under fine-grained conditions has not been investigated.

However, visual, in situ observations of grout diffusion within coarse-grained materials, particularly those containing large particles, are still lacking. Particularly concerning the dynamic effects of fine-particle migration and clogging on grout diffusion pathways, existing studies have not fully achieved real-time observation and quantitative characterization. Therefore, the primary objectives of this study are to: (1) visually investigate the influence of fine-particle content (no, partial, and complete fine-particle loss) on grout diffusion patterns and reinforcement efficiency in coarse-grained materials using transparent soil technology; (2) quantify the diffusion process under constant pressure (40 kPa) via PIV; and (3) elucidate the underlying mechanisms governing grout diffusion, particularly the coupled effects of fine-particle migration, clogging, and grout viscosity. This work aims to bridge the gap between macroscopic grouting outcomes and microscopic particle-scale processes, providing insights for more reliable grouting design in dam rehabilitation.

## Model test design

### Test apparatus

In actual engineering applications, the required pressure for permeation grouting ranges from 0.1 to 0.4 MPa. Based on similarity relationships, the calculated pressure for laboratory grouting tests was to be 10–60 kPa. At 10 kPa grouting pressure, the grout fails to effectively diffuse within the model. Conversely, applying 60 kPa grouting pressure can remarkably shorten the grouting duration. Furthermore, grout diffusion in mixtures lacking fine particles and uniformly coarse-grained mixtures is dominated by gravity at 20 kPa. Hence, a grouting pressure of 40 kPa was selected for the simulation.

**Fig 1:**
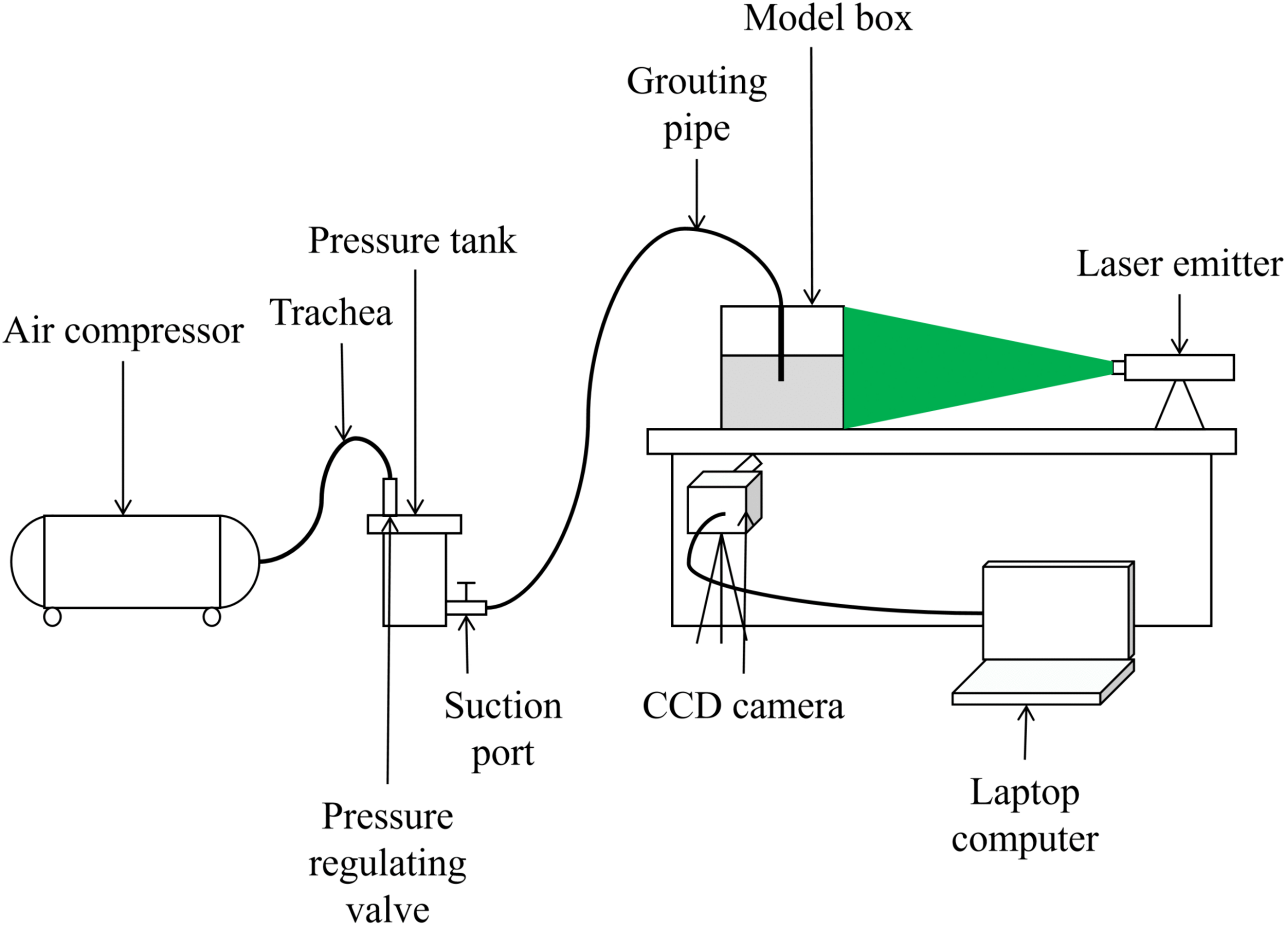
Physical diagram of grouting device.

To more realistically simulate the actual grouting process, a custom-built constant-pressure grouting device was used in the grouting test to inject grout into the model at a constant pressure. The grouting apparatus consists of an air compressor, constant-pressure valve, pressure barrel, and grouting pipe. Pressurized gas from the air compressor is regulated by the constant-pressure valve to the required pressure before being introduced into the pressure barrel. When the valve of the pressure barrel is opened, the grout inside is expelled under constant pressure. Then it flows through the grouting pipe into the model box. The physical model is illustrated in Fig 1.‌‌

### Model scale calculation

**Fig 2:**
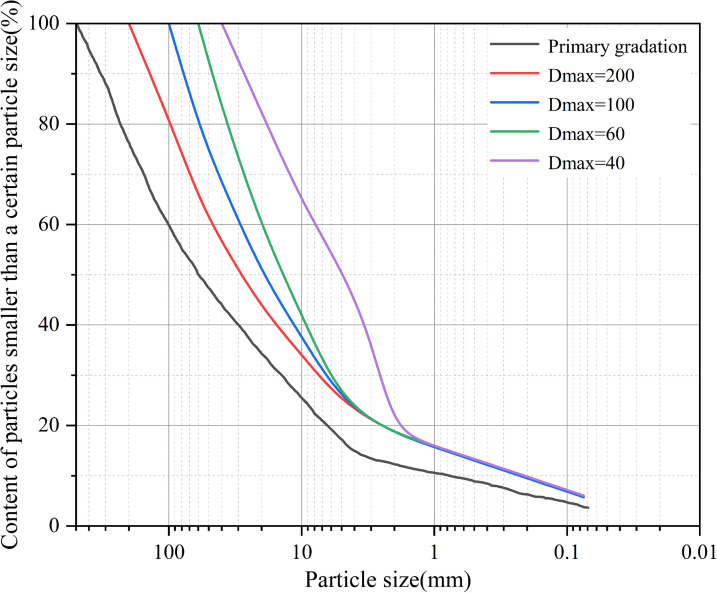
Original Gradation and Gradation Curve After Scale Reduction from Literature.

Previous researchers [16] have analyzed the particle-size scaling of coarse-grained materials and their mechanical properties before and after particle scaling in the Dashixia Water Conservancy Project. Therefore, the Dashixia Water Conservancy Project was selected as the actual engineering case in our study. In the actual project, the maximum particle size of the rockfill material is 500 mm, which far exceeds the maximum size permitted in laboratory tests. Particle-size scaling was carried out according to the method reported in the literature, and the obtained gradation results were compared with those reported in previous studies and were found to be similar to those reported in the literature, as illustrated in Fig 2. The rockfill material adopted in the actual project consists of irregular blocks with angular particles. Additionally, fused quartz particles were selected as the transparent solid particles to maintain geometric similarity with the actual rockfill material in laboratory testing [17,18]. Their irregular shape helped simulate the angular rockfill structure.

In this study, the similarity gradation method and the equivalent replacement method were employed to scale the actual coarse-grained material. The minimum internal dimension of the model box was five times the maximum particle size. Using the maximum size of 60 mm permitted in laboratory tests would necessitate a substantial quantity of material, leading to an oversized model box. Therefore, the maximum particle size was reduced to 40 mm. Similarity calculations were performed with a similarity table, and the similar gradation method was adopted to ensure gradation similarity of the model material. Nonetheless, the mass fraction of fine particles in the model was maintained by the equivalent replacement method to account for the elatively high fine-proportion after scaling. Particle scaling calculations were conducted according Equation (1). Model similarity calculations were also completed, with the similarity calculation table presented in Table 1.

**Table 1:** Similarity Relationship Calculation Table.

physical quantity	Similarity formula	prototype	Model	Similarity coefficient
High(H)	$C_{H}=\frac{C_{prototype}}{C_{model}}$	247 m	1 m	247
Length(L)	$C_{L}=\frac{C_{prototype}}{C_{model}}$	25mm	8mm	3.13
Density(ρ)	$C_{\rho }=\frac{\rho_{prototype}}{\rho_{model}}$	1.94 kg/m^3^	1.25 kg/m^3^	1.55
Viscosity coefficient(ν)	$C_{\nu }=\frac{\rho_{prototype}}{\rho_{model}}$	5010mPa·s	1600mPa·s	3.13


$$\begin{array}{c} \begin{array}{c} P_{i}=\frac{P_{oi}}{P_{\text{5}}-P_{dmax}}P_{\text{5}} \end{array} \end{array}$$ (1)

where $P_{oi}$ denotes the original gradation content of a certain particle size group, %; $P_{dmax}$ denotes the content of oversized particles, %; $P_{i}$ denotes the content of coarse particles after replacement, %; and $P_{5}$ denotes the original gradation content of particles larger than 5 mm, %.

### Selection of test materials

#### Solid particles.

**Fig 3:**
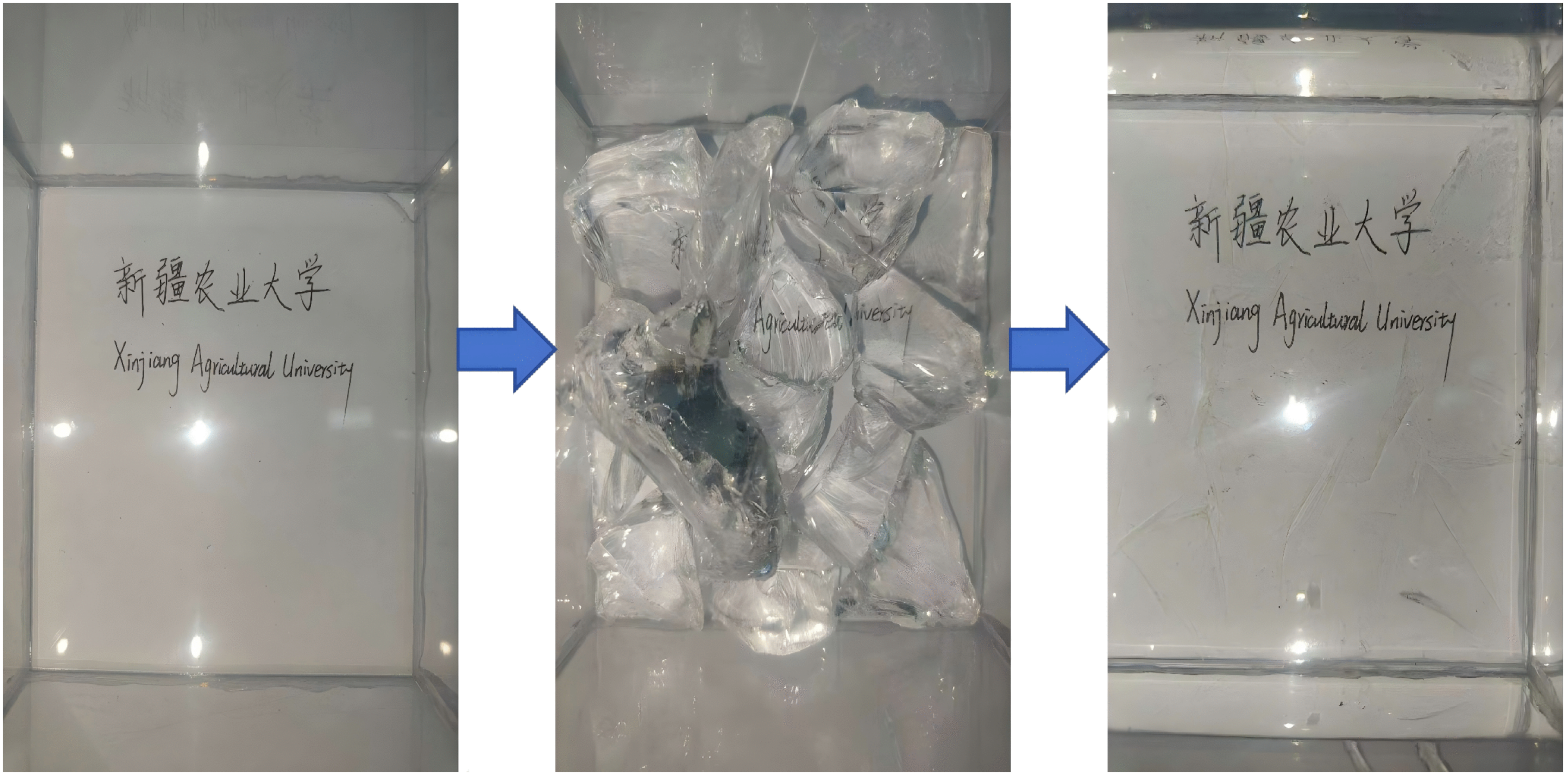
Model comparison diagram showing whether to add pore fluid or not.

To achieve visual measurements in transparent soil tests, this study selected fused quartz sand as the skeleton material. Its main advantages are that fused quartz is a non-crystalline SiO_2_ with a stable refractive index and isotropy. After the refractive index is matched, scattering at the solid-liquid interface is significantly reduced, thereby achieving high transparency and meet the optical measurement requirements of PIV/PLIF (Fig 3). At the same time, it has high hardness and good wear resistance, and is not prone to breakage or the generation of fine particles under long-term immersion and seepage conditions, thereby maintaining the stability of the pore structure; in addition, fused quartz has strong chemical inertness and good compatibility with commonly used refractive index-matching liquid systems, which can reduce the risk of turbidity caused by reactions, precipitation, and contamination.

#### Pore fluid.

**Fig 4:**
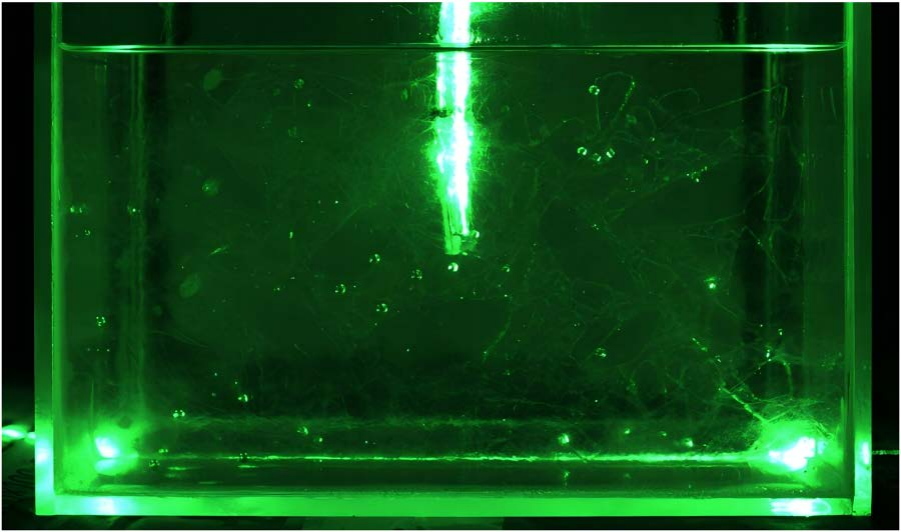
Transparent soil model under laser irradiation.

In the absence of pore fluid, the fused quartz sand model appears opaque under laser illumination, restricting observation to the fused quartz particles located near the chamber walls. Considering that internal grouting changes cannot be effectively observed under this condition, a pore fluid was prepared from No. 15 white mineral oil and n-dodecane at a mass ratio of 0.8:1. After preparation, the refractive index was measured using an Abbe refractometer, yielding a refractive index of 1.4585 for the prepared pore fluid [19,20]. In addition, the prepared pore fluid was injected to minimize bubble interference within the model. Subsequently, solid particles were placed in layers to build the model [21], as displayed in Fig 4.

### Grouting material

**Fig 5:**
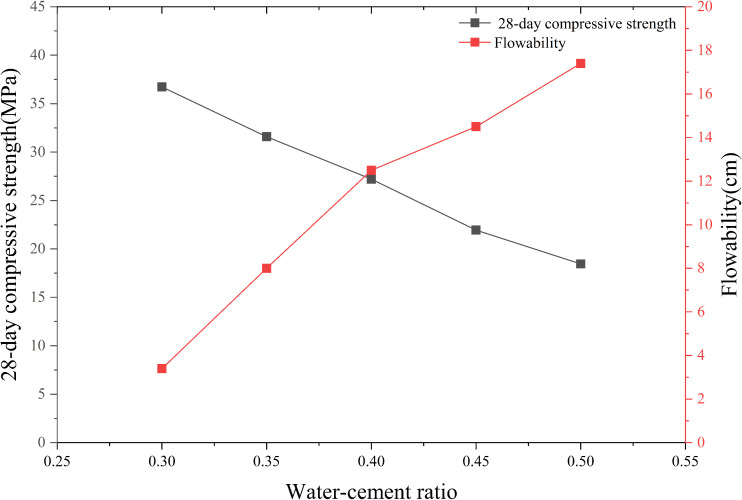
Slurry ratio comparison parameters.

P.O 42.5R ordinary Portland cement slurry was employed in this study as the actual grout slurry. Flow tests were conducted at water-to-cement ratios of 0.30, 0.35, 0.40, 0.45, and 0.50, alongside compressive strength tests at curing ages of 3, 7, and 28 days. The test results are depicted in Fig 5.

After comparative evaluation, cement slurry with a water-to-cement ratio of 0.4 was selected as the grouting slurry. Subsequently, viscosity measurements were conducted on the cement slurry with a water-to-cement ratio of 0.4. A transparent grout slurry with similar apparent viscosity was employed to simulate the cement slurry because the cement slurry remains opaque during grouting tests in transparent soil. Rotational viscosity measurements of the two-component epoxy resin adhesive revealed that its rotational viscosity was comparable to that of the cement slurry with a water-to-cement ratio of 0.4 after similarity conversion calculations. Hence, the two-component epoxy resin adhesive was utilized for the grouting tests.

**Fig 6:**
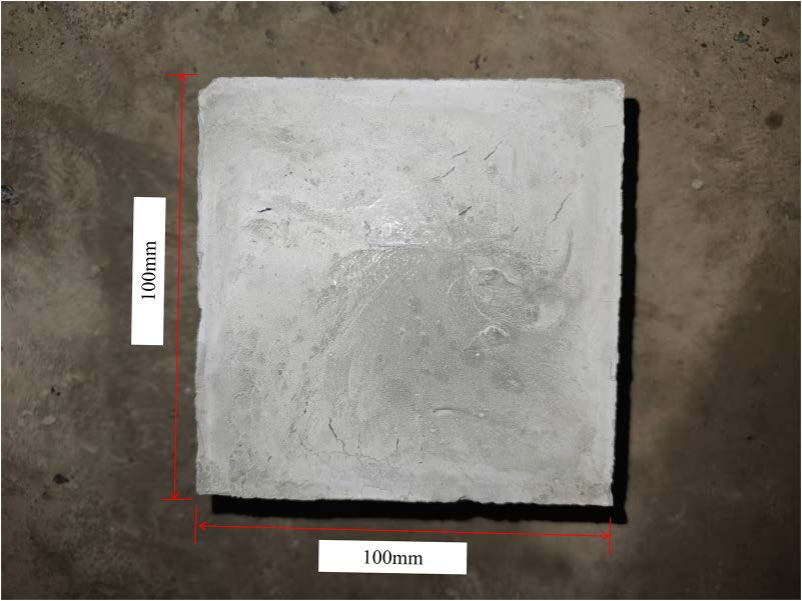
Epoxy Resin and Cement Slurry Shrinkage Test Specimens.


In additon, shrinkage tests were conducted on both epoxy resin and cement paste. Cuboid specimens measuring 100 × 100 × 50 mm were prepared. The shrinkage test specimens are shown in Fig 6. The initial height of the cement paste specimen was 50 mm. After 28 days of curing and shrinkage, both each specimen measured 49.5 mm in height. Considering that only height changes were measured, the linear shrinkage equation (2) was applied for calculation, yielding a shrinkage rate of 1% for both specimens.

$$\begin{array}{c} \begin{array}{c} S_{t}=\frac{L_{\text{0}}-L_{f}}{L_{\text{0}}}\times \text{100}\% \end{array} \end{array}$$ (2)

where $S_{t}$ denotes linear shrinkage rate, ％; $L_{0}$ denotes the initial length, mm; $L_{f}$ denotes the final length, mm.

Since the two-component epoxy resin adhesive is colorless and transparent during grouting, a visible spectrophotometer was used with air and water as blank controls. At the 532 nm wavelength of the laser source, transmittance ratio measurements were conducted on pore fluid with a refractive index of 1.4585 and epoxy resin. The results show that the difference in transmittance ratio between the pore fluid and epoxy resin was only 3.57%. In other words, without added dyes, the epoxy resin has transparency comparable to that of the transparent soil model with a refractive index of 1.4585.

The diffusion process of the grout simulant could not be clearly identified during post-processing of experimental images using PIV software. Therefore, Rhodamine B dye was added to the epoxy resin for visualization. The epoxy resin containing the dye appeared orange-yellow under laser irradiation. After the experiment, the epoxy-resin-injection area appeared pink under natural light, allowing the bonding and dispersion characteristics of the grout simulant to be identified.

### Trial protocol

**Fig 7:**
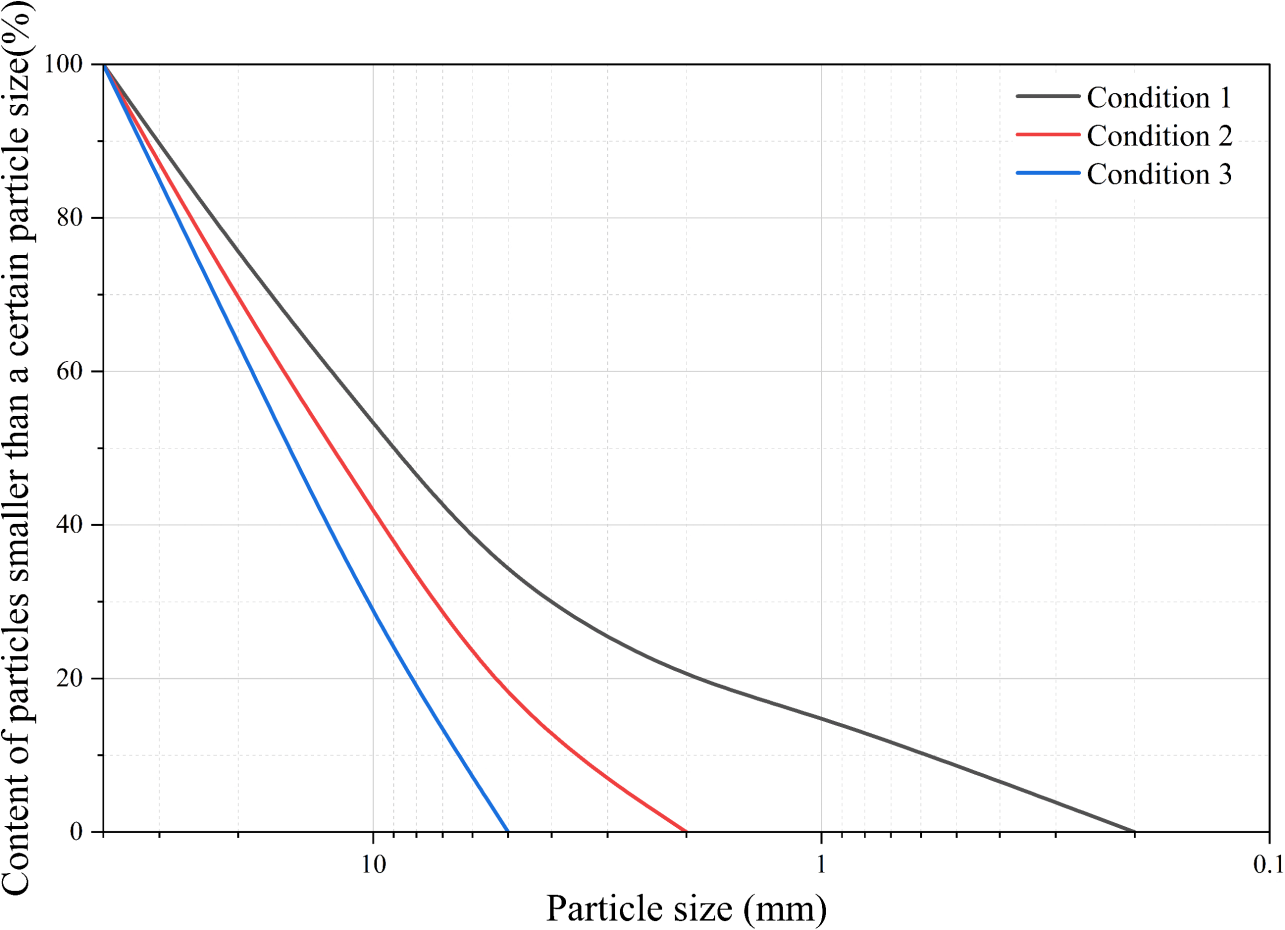
Gradation Curves for Different Operating Conditions.

With the purpose of investigating the effect of fine-particles on the grouting reinforcement of coarse-grained materials, model specimens were constructed with varying fine-particle contents: complete graded after scaling, partial fine-particles loss after scaling, and completely lacking fine-particles. The gradation curves are displayed in Fig 7.

### Test procedure

#### Transparent soil grouting experiment.

(1) The pore fluid was first prepared. After its refractive index was determined to be 1.4585, it was injected into the model box with internal dimensions of 200 × 190 × 190 mm.
(2) The model was filled layer by layer with fused quartz sand following the working condition gradation. After each layer was placed, the model was evacuated until no bubbles remained in the soil specimen.
(3) The laser was turned on, with the laser sheet aligned with the central cross section the model, and the CCD camera was mounted to capture images during grouting.
(4) The grouting apparatus was assembled, and its airtightness was verified.
(5) A total 500 g of grout simulant was prepared and poured into the pressure vessel; the vessel was sealed with its valve closed.
(6) The air compressor was turned on, and the pressure regulator on the pressure vessel was simultaneously adjusted to maintain an injection pressure of 40 kPa.
(7) The pressure vessel valve was opened to inject the grout into the model box. Simultaneously, the CCD camera was used to capture the grout diffusion process at a rate of 1 frame per second until all prepared grout was injected into the model. The grouting duration was 300 seconds. Three replicate experiments were conducted under identical conditions to minimize measurement error.

#### Cementitious body collection and permeability test.

After testing was completed, core samples were extracted from the cemented material using a core drill. The drill bit had an inner diameter of 110 mm, with drilling positions centered on the area around the grout pipe. Following sampling, falling-head permeability tests were conducted. Under a hydraulic head of 1-meter, the core sample was placed inside a 1-meter-long PVC pipe. The annular gap between the core sample and the PVC pipe as well as the side surfaces of the core, were sealed with polytetrafluoroethylene tape to ensure that water flowed only through the specimen from the bottom. The permeability coefficient was calculated using Equation (3).


$$\begin{array}{c} \begin{array}{c} K=\frac{aL}{At}ln\frac{{\text{h}}_{\text{1}}}{{\text{h}}_{\text{2}}} \end{array} \end{array}$$ (3)

where K denotes the permeability coefficient, cm/s; a represents the cross-sectional area of the water pipe, cm^2^; A denotes the cross-sectional area of the sample, cm^2^; L denotes the length of the sample, cm; t denotes the time it takes for the water level to drop from $h_{1}$ to h2, s;$h_{\text{1}}$ and $h_{\text{2}}$ denote the initial and final hydraulic head differences, cm.

## Analysis of grouting results for coarse-grained materials with different fine-grained contents

### Comparative analysis of slurry diffusion processes

**Fig 8:**
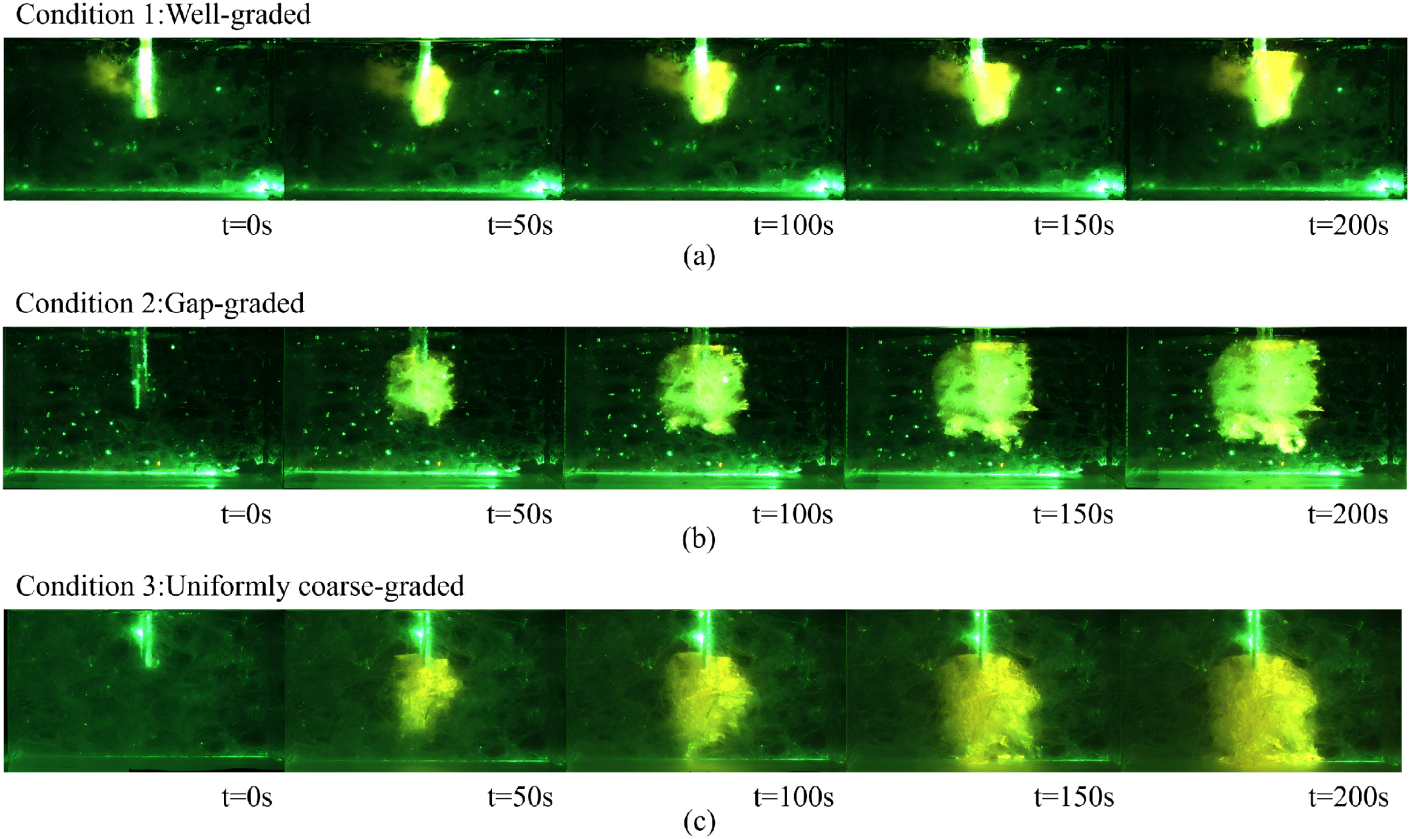
Slurry Diffusion under Various Operating Conditions: (a) Condition 1 (b) Condition 2 and (3) Condition 3.

Under a grout viscosity of 1600 mPa·s and an injection pressure of 40 kPa, the diffusion behavior of the grout under three test conditions is illustrated in Fig 8. As shown in the figure, the grout diffusion exhibits characteristics similar to spherical diffusion.

Operating scenario 1 involves a higher proportion of fine-particles, leading to a smaller grout simulant diffusion range, as presented in Fig 8(a). Operating scenario 2 contains fewer fine particles, causing the slurry to demonstrate spherical diffusion with nearly identical vertical and horizontal diffusion ranges, as illustrated in Fig 8(b). In operating scenario 3, the absence of fine particles brings about larger inter-particle voids and thus a broader slurry diffusion range. Nevertheless, the slurry diffuses more rapidly in the vertical direction due to gravitational influence, as depicted in Fig 8(c).

**Fig 9:**
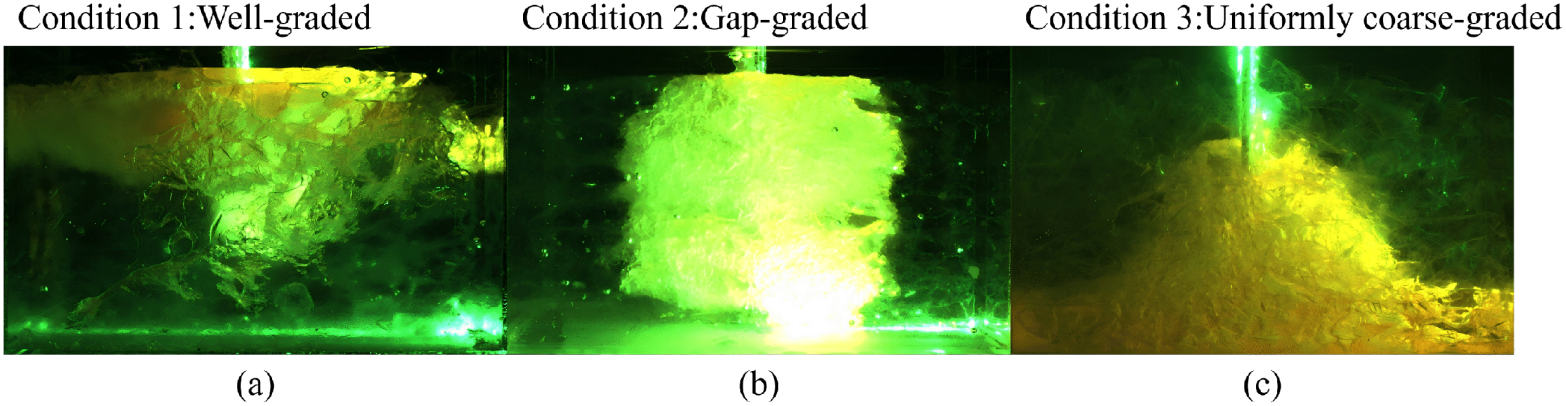
Grout diffusion state after grouting completion: (a) Condition 1 (b) Condition 2 and (3) Condition 3.

During the grouting process, the grouting materials in Scheme 1 and Scheme 2 spread upward under pressure towards the grouting pipe, while the grouting material in Scheme 3 diffused vertically but did not surround the grouting pipe. After the grouting was completed, the grouting material in Scheme 1 encountered diffusion resistance and overflowed from the grouting port, spreading on the model surface. Its diffusion behavior was similar to that reported in Reference [15] (Fig 9(a)). The grouting material in Scheme 2 diffused downward to the bottom of the model and then began to spread horizontally, as shown in Fig 9(b). The grouting material in Scheme 3 flowed through the pores between particles and accumulated at the bottom of the model, as shown in Fig 9(c).

### Analysis of grouting processes under different operating conditions

**Fig 10:**
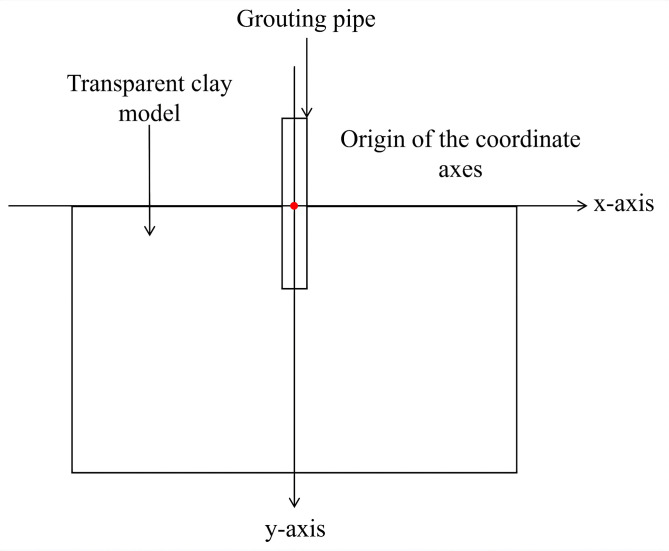
Two-Dimensional Coordinate System of the Grouting Model.

To better describe the diffusion changes of grout during the grouting process, the x-axis is defined along the top of the model, the positive y-direction as the direction of the grouting pipe, and the origin of the coordinate axes as the point where the top of the model contacts the grouting pipe (Fig 10). In Particularly, the diffusion range at the bottom of the model is greater than the horizontal diffusion distance of grout in the middle section of the model. Thus, measurements were taken 20 mm above the bottom of the model to better describe the horizontal diffusion range of grout in the middle section of the model.

### Analysis of grouting results for operating condition 1

**Fig 11:**
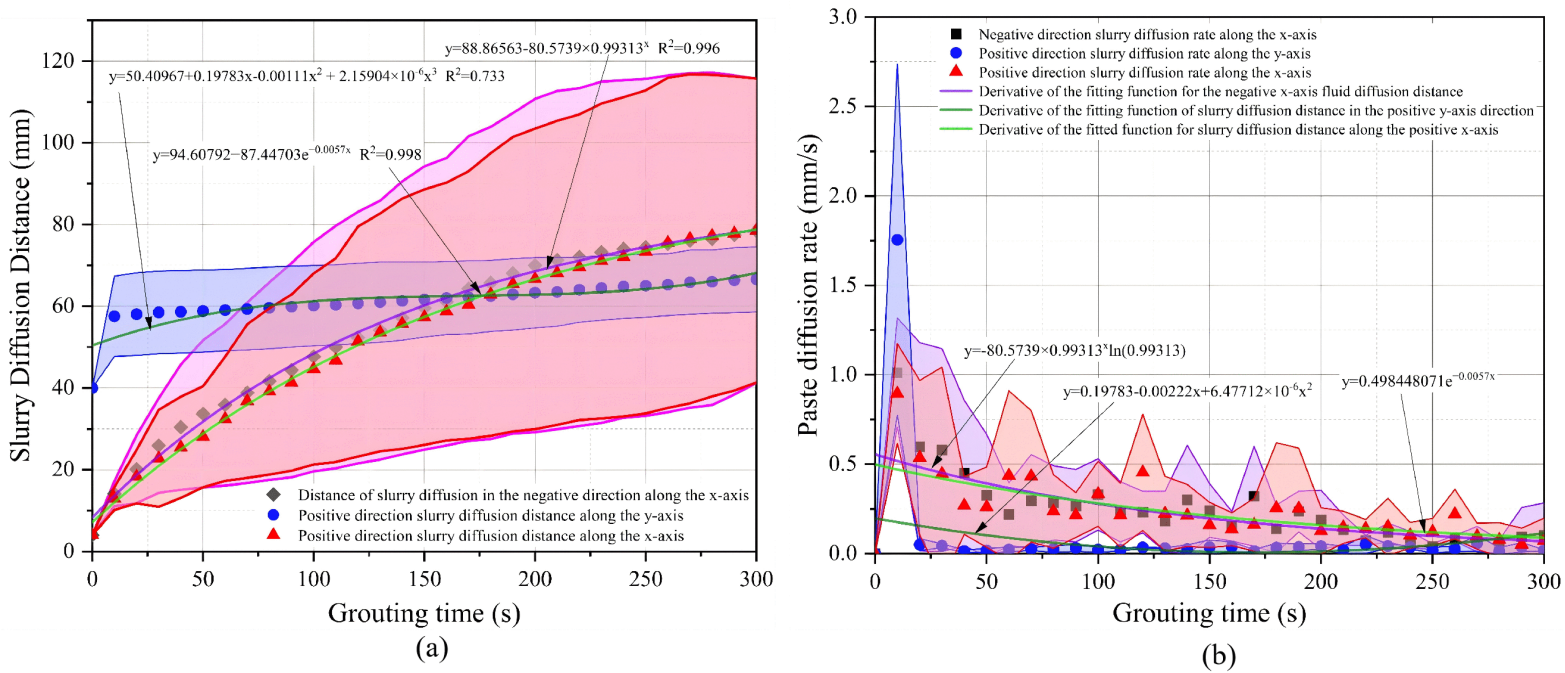
Operating Condition 1 Slurry Diffusion Range and Slurry Diffusion Rate: (a) Condition 1 Slurry Diffusion Range (b) Slurry Diffusion Rate.

In Condition 1, grout simulant diffusion exhibited a pronounced lateral preference. As shown in Fig 11(a) and Table 2, at 300 s, the final diffusion distances in the negative x-, positive x-, and positive y-directions were 78.49 ± 37.24 mm, 78.54 ± 37.17 mm, and 66.59 ± 7.96 mm, respectively. The average lateral diffusion distance was approximately 78.52 mm, which was significantly greater than the vertical diffusion distance. This suggests that under a high fine-particle content, vertical penetration of the grout simulant was more strongly constrained, causing the diffusion path to shift laterally. Once the pore spaces between particles were filled, further advancement of the grout simulant became difficult. Under the action of injection pressure, the grout simulant migrated through the pores between the injection pipe wall and the surrounding particles toward the model surface, where it flowed laterally while continuing to infiltrate the model interior. Because the large fused-quartz particles in the model were randomly distributed, the fine particles were unevenly distributed within the pores between the coarse particles, leading to greater variability among the replicate tests. The one-way ANOVA p-values for the negative x-, positive x-, and positive y-directions were 1.147 × 10^-15^, 3.554 × 10^-13^, and 1.425 × 10^-22^, respectively. The corresponding Tukey groupings were A/A/B, A/A/B, and A/B/C, indicating significant differences among the three replicate tests under this condition; in particular, repeatability in the vertical direction was relatively poor. These results suggest that, under a high fine-particle content, slurry diffusion is more sensitive to local medium heterogeneity. As shown in Fig 11(b) and Table 2, the peak diffusion rates in the three directions occurred during the initial stage of injection and were 1.01 ± 0.30 mm/s, 0.90 ± 0.28 mm/s, and 1.76 ± 0.98 mm/s, respectively. This indicates that when the grout simulant first entered the model, it advanced rapidly under the combined effects of injection pressure and gravity, despite confinement within the pore space. Subsequently, the uneven distribution of fine particles slowed grout simulant diffusion. After reaching the model surface, the slurry flowed along the surface while continuing to diffuse into the model interior, resulting in large fluctuations in the horizontal direction. This suggests that the early downward infiltration did not persist, but was later limited by pore-throat constriction and local blockage. Overall, Condition 1 was characterized by enhanced pore-throat constriction caused by the high fine-particle content. After a short period of rapid initial infiltration, the grout simulant gradually shifted to lateral diversion and surface spreading, ultimately forming a diffusion pattern characterized by insufficient vertical penetration, enhanced lateral diffusion, and poor repeatability.

**Table 2:** Statistical summary of grout diffusion distance and diffusion rate for Condition 1.

Indicators	The negative x direction	Positive x direction	Positive y direction
Final diffusion distance (mm)	78.49 ± 37.24	78.54 ± 37.17	66.59 ± 7.96
One-way ANOVA (P value)	1.147 × 10^-15^	3.554 × 10^-13^	1.425 × 10^-22^
Tukey grouping	A/A/B	A/A/B	A/B/C
Peak diffusion rate (mm/s)	1.01 ± 0.30	0.90 ± 0.28	1.76 ± 0.98

Note: Data are presented as mean ± standard deviation (SD), with n = 3 independent replicates. The P values of one-way ANOVA were obtained from the analysis of variance of the three replicate tests in the same direction. Tukey grouping was derived from Tukey’s post hoc multiple comparison test. Identical letters indicate no significant difference among groups, whereas different letters indicate significant differences (p < 0.05).

### Analysis of grouting results for operating condition 2

**Fig 12:**
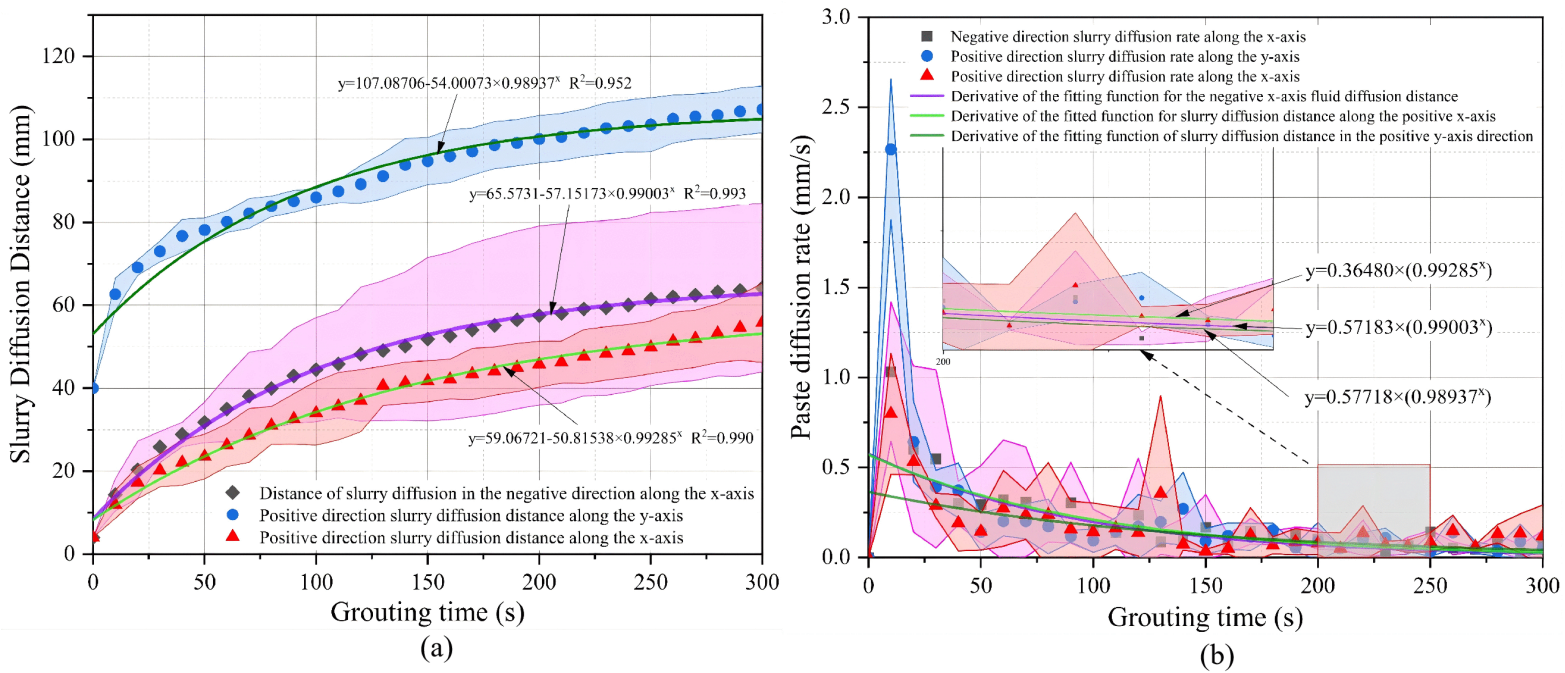
Operating Condition 2 Slurry Diffusion Range and Slurry Diffusion Rate: (a) Slurry Diffusion Rang (b) Slurry Diffusion Rate.

Condition 2 exhibited a more coordinated diffusion pattern than Condition 1. As shown in Fig 12(a) and Table 3, the final diffusion distances in the negative x-, positive x-, and positive y-directions were 64.23 ± 20.30 mm, 55.79 ± 9.54 mm, and 107.22 ± 5.64 mm, respectively. Compared with Condition 1, the diffusion distance in the positive y-direction increased significantly, while noticeable lateral spread was still maintained. This indicates that an appropriate fine-particle content not only weakened the rapid channeling effect of large pores, but also did not cause severe blockage, thereby facilitating the formation of a more continuous diffusion front. The one-way ANOVA p-values for the three directions were 0.911, 0.007, and 0.094, respectively, and the corresponding Tukey groupings were A/A/A, A/B/B, and A/A/A. These results indicate that no significant differences were observed among the repeated experiments in the negative x- and positive y-directions, suggesting good repeatability in these two directions, whereas some fluctuation remained in the positive x-direction. This suggests that although Condition 2 was not completely stable in all directions, it generally exhibited higher repeatability and better diffusion controllability than Condition 1. As shown in Fig 12(b) and Table 3, the peak diffusion rates in the three directions were 1.03 ± 0.39 mm/s, 2.27 ± 0.39 mm/s, and 0.80 ± 0.34 mm/s, respectively. Although the peak diffusion rate was highest in the positive x-direction, the final diffusion distance remained greatest in the positive y-direction. This suggests that local lateral branches may have formed short-lived rapid pathways during the early stage, whereas the overall diffusion behavior was still characterized by enhanced vertical development and spatial coordination. Overall, this condition indicates that an appropriate fine-particle content optimized the pore structure formed by coarse particles, restrained excessively rapid vertical channeling through large pores, and at the same time did not hinder lateral diffusion of the grout simulant. As a result, the grout simulant exhibited more favorable filling and diffusion characteristics in all directions.

**Table 3:** Statistical summary of grout diffusion distance and diffusion rate for Condition 2.

Indicators	The negative x direction	Positive x direction	Positive y direction
Final diffusion distance (mm)	64.23 ± 20.30	55.79 ± 9.54	107.22 ± 5.64
One-way ANOVA (P value)	0.911	0.007	0.094
Tukey grouping	A/A/A	A/B/B	A/A/A
Peak diffusion rate (mm/s)	1.03 ± 0.39	2.27 ± 0.39	0.80 ± 0.34

Note: Data are presented as mean ± standard deviation (SD), with n = 3 independent replicates. The P values of one-way ANOVA were obtained from the analysis of variance of the three replicate tests for the same diffusion direction. Tukey grouping were derived from Tukey’s post hoc multiple comparison test. Identical letters indicate no significant difference among groups, whereas different letters indicate significant differences (p < 0.05).

### Analysis of grouting results for operating condition 3

**Fig 13:**
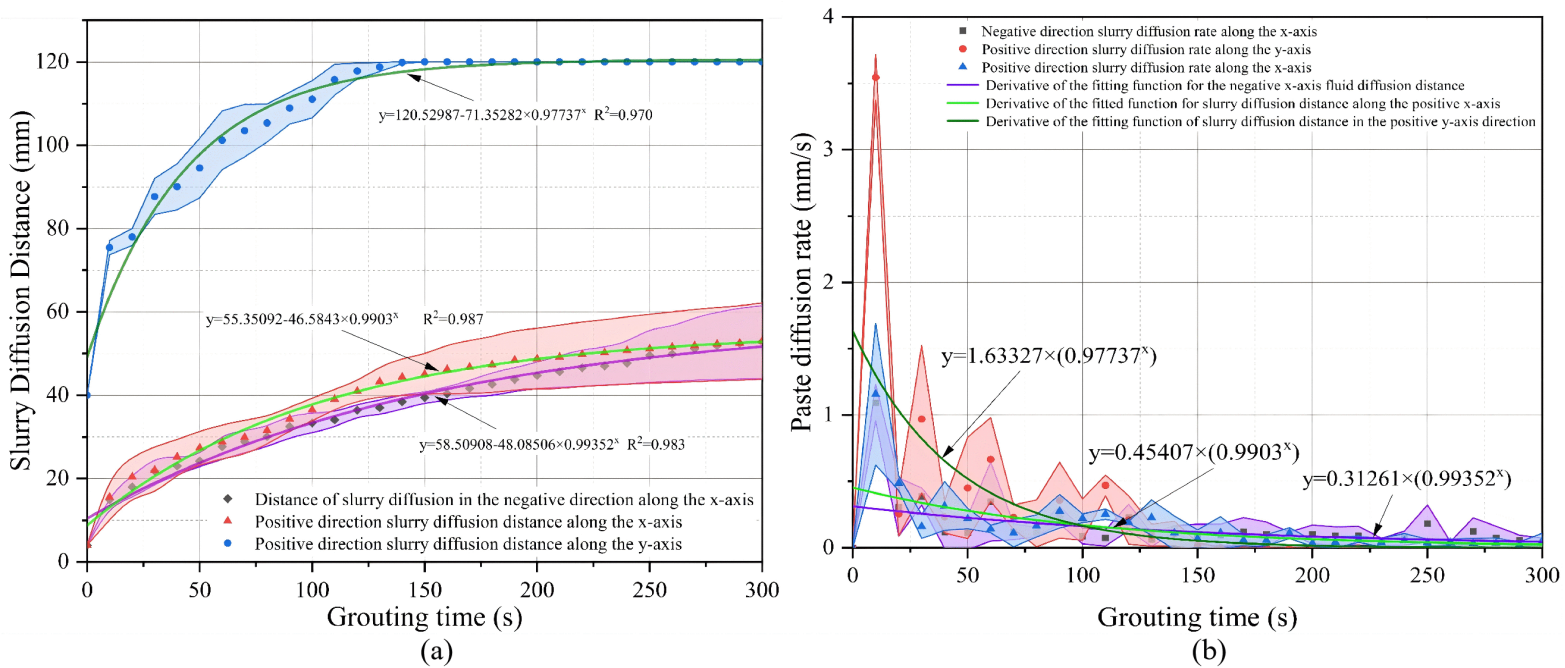
Operating Condition 3 Slurry Diffusion Range and Slurry Diffusion Rate: (a) Slurry Diffusion Range (b) Slurry Diffusion Rate.

Condition 3 exhibited the most pronounced vertical diffusion dominance among the three conditions. As shown in Fig 13(a) and Table 4, the final diffusion distances in the negative x-, positive x-, and positive y-directions were 52.80 ± 8.71 mm, 53.01 ± 9.19 mm, and 120.00 ± 0.00 mm, respectively. The diffusion distance in the positive y-direction was approximately 2.27 times the average lateral diffusion distance, while the diffusion distances in the negative x- and positive x-directions were nearly identical. This indicates that under this condition, the grout simulant primarily percolated rapidly in the vertical direction, whereas lateral spreading remained relatively limited. The one-way ANOVA p-values for the three directions were 0.310, 0.005, and 0.836, respectively, and the corresponding Tukey groupings were A/A/A, A/AB/B, and A/A/A. These results indicate that no significant differences were observed among the repeated experiments in the negative x- and positive y-directions, whereas some variability remained in the positive x-direction. In particular, the diffusion distance in the positive y-direction was 120.00 ± 0.00 mm, indicating that the slurry reached the bottom of the model in all repeated tests. By contrast, although some fluctuations were still observed in the positive x-direction, the fluctuation range was significantly smaller than that in the other two conditions. As shown in Fig 13(b) and Table 4, the peak diffusion rates in the three directions were 1.09 ± 0.14 mm/s, 1.16 ± 0.53 mm/s, and 3.54 ± 0.17 mm/s, respectively, with the vertical peak rate being significantly higher than the lateral rates. These results suggest that, in the absence of fine-particle constraints, the connectivity of large pores was enhanced, allowing the grout simulant to percolate rapidly along low-resistance vertical channels during the initial stage of grouting. In contrast, lateral diffusion remained limited. Overall, the physical mechanism of Condition 3 can be interpreted as follows: without fine particles, the continuity of large pores was enhanced, causing the simulant to migrate preferentially and rapidly in the vertical direction. As a result, the simulant exhibited the strongest downward penetration capacity and relatively high vertical repeatability, whereas its lateral wrapping and uniform filling capacities were comparatively insufficient.

**Table 4:** Statistical summary of grout diffusion distance and diffusion rate for Condition 3.

Indicators	The negative x direction	Positive x direction	Positive y direction
Final diffusion distance (mm)	52.80 ± 8.71	53.006 ± 9.19	120 ± 0
One-way ANOVA (P value)	0.310	0.005	0.836
Tukey grouping	A/A/A	A/AB/B	A/A/A
Peak diffusion rate (mm/s)	1.09 ± 0.14	1.16 ± 0.53	3.54 ± 0.17

Note: Data are presented as mean ± standard deviation (SD), with n = 3 independent replicates. The P values were obtained by one-way ANOVA for the three replicate tests in the same direction. Tukey grouping was derived from Tukey’s post hoc multiple comparison test. Identical letters indicate no significant difference among groups, whereas different letters indicate significant differences (p < 0.05).

### Cross-condition statistical comparison

A comparison of Tables 2–4 shows clear differences among the three conditions in terms of diffusion direction, diffusion stability, and peak diffusion rate. A comparison of the final diffusion distances shows that Condition 1 exhibited the most pronounced lateral diffusion, with the negative x- and positive x-direction distances reaching 78.49 ± 37.24 mm and 78.54 ± 37.17 mm, respectively, whereas the distance in the positive y-direction was only 66.59 ± 7.96 mm. This indicated that under a high fine-particle content, the grout simulantwas more likely to spread laterally. By contrast, in Condition 2, the final diffusion distance in the positive y-direction increased to 107.22 ± 5.64 mm, while the lateral diffusion distances remained 64.23 ± 20.30 mm and 55.79 ± 9.54 mm, indicating that this condition achieved a balance between downward diffusion and lateral filling. Condition 3 exhibited the strongest vertical penetration, with the final diffusion distance in the positive y-direction reaching 120.00 ± 0.00 mm, whereas the lateral diffusion distances on both sides were only about 53 mm. This indicated that in the absence of fine-particle constraints, the grout simulant preferentially percolated downward through large vertical pores. A comparison of the peak diffusion rates showed that the peak diffusion rates in the positive y-direction under the three conditions were 1.76 ± 0.98 mm/s, 0.80 ± 0.34 mm/s, and 3.54 ± 0.17 mm/s, respectively. This showed that Condition 3 had the strongest early vertical advancement capacity, followed by Condition 1, whereas Condition 2 had the lowest peak rate in the positive y-direction. Meanwhile, the peak diffusion rate in the positive x-direction under Condition 2 reached 2.27 ± 0.39 mm/s, which was higher than those under the other two conditions, indicating that local channel switching was more pronounced under a moderate fine-particle content, although this did not alter the overall relatively balanced diffusion pattern. A comparison of repeatability showed that the one-way ANOVA p-values for Condition 1 in the negative x-, positive x-, and positive y-directions were all well below 0.05, and the Tukey groupings differed significantly, indicating relatively high dispersion among the replicate tests. In Condition 2, no significant differences were observed among the repeate tests in the negative x- and positive y-directions, whereas some fluctuation remained in the positive x-direction. Condition 3 likewise exhibited good repeatability in the negative x- and positive y-directions, and the final diffusion distance in the positive y-direction was 120.00 ± 0.00 mm, indicating a highly stable main vertical diffusion pathway. Taken together with Tables 2–4 and Figs 11–13, these results suggest that the main differences among the three conditions were not merely reflected in the magnitude of the diffusion range, but rather in the distinct pore-structure control mechanisms induced by variations in fine-particle content: Condition 1 was characterized by stronger pore-throat constriction, Condition 2 exhibited the most coordinated diffusion pathway, and Condition 3 developed the most pronounced vertical low-resistance channels.

### Comparison of stone body morphology

**Fig 14:**
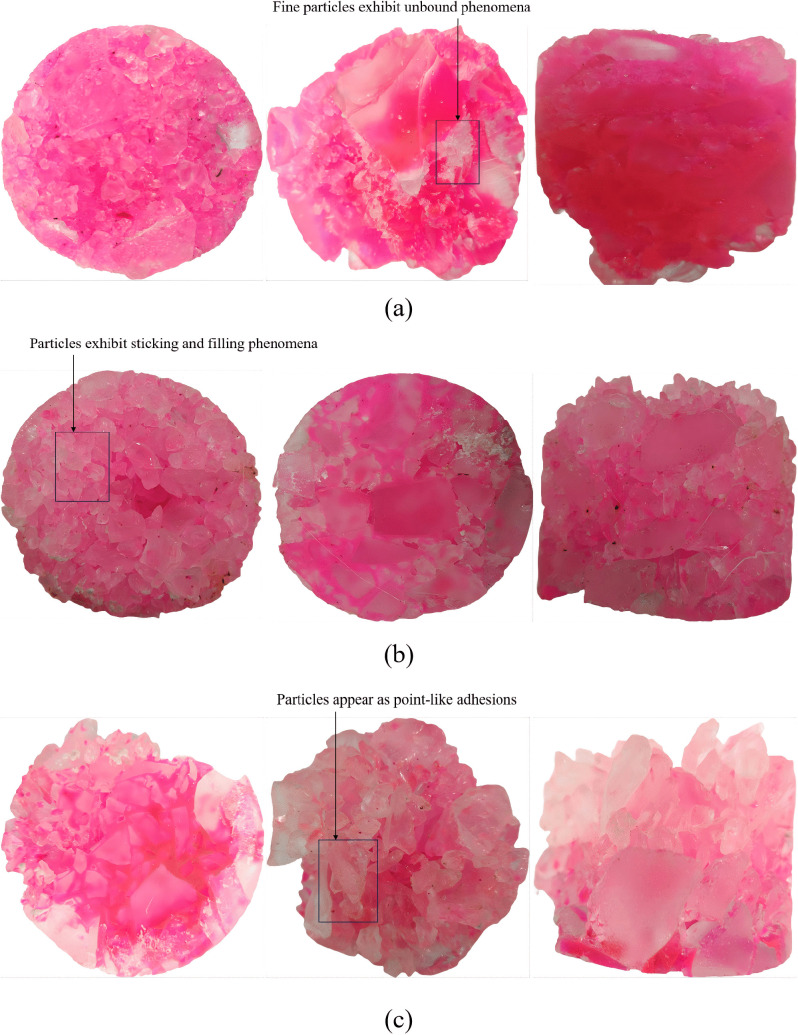
Core samples of the cemented structure under different conditions: (a) Condition 1 (b) Condition 2 (c) Condition 3.

The three types of core samples with different gradations indicate that the gradation determines the migration and consolidation pattern of the grout simulant within the porous framework (Fig 14). Among them, the 5–40 mm particle-size range froms relatibely large pore channels are relatively large, allowing the grout simulant to enter easily but not to remain effectively. The cemented bodies are mainly connected by point-like bridges and locally coated. The structural uniformity and continuous filling capacity are relatively limited (Fig 14(a)). The 2–40 mm size range can significantly reduce the particle pores when injecting the grout simulant and achieve continuous consolidation. However, it is also relatively prone to the bridging effect, causing premature blockage in local areas. As a result, in areas with more fine particles, there are phenomena of insufficient bonding or weak bonding ability. The overall uniformity and integrity of the cemented bodies in this grading are better (Fig 14(b)). The 0.2–40 mm size range has a high content of fine particles. During the injection process, the grout simulant can only penetrate downward to a limited extent, causing premature retention of the grout simulant in this region. After that, the simulant flows freely on the model surface and naturally infiltrates the model under the influence of gravity, resulting in the particles below the injection pipe not being effectively consolidated. The effectively penetrated part has the best particle bonding effect, but the small pores prevent the simulant from being fully filled, leaving the internal fine particles in a granular state (Fig 14(c)).

### Permeability coefficient

**Fig 15:**
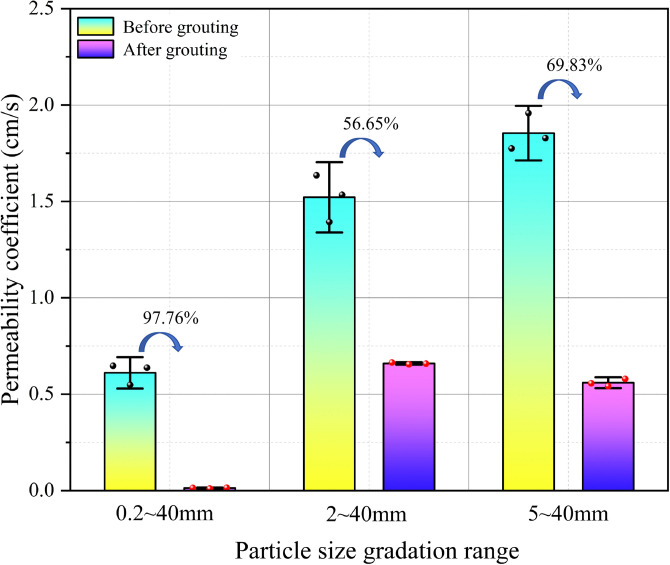
Comparison of permeability coefficients before and after grouting in different particle size ranges.

As shown in Fig 15 and Table 5, the permeability coefficients of the three gradations decreased statistically significantly after grouting. Before grouting, the permeability coefficients of the 0.2–40 mm, 2–40 mm, and 5–40 mm gradations were 0.61 ± 0.054 cm/s, 1.52 ± 0.12 cm/s, and 1.85 ± 0.09 cm/s, respectively; after grouting, they decreased to 0.01 ± 0.002 cm/s, 0.66 ± 0.004 cm/s, and 0.56 ± 0.02 cm/s, corresponding to reduction rates of 97.76%, 56.65%, and 69.83%, respectively. Moreover, the p-values for the comparisons before and after grouting were 0.0027, 0.0065, and 0.0012, respectively, all of which were less than 0.05, indicating that grouting had a statistically significant effect on reducing the permeability coefficient. Among the three gradations, the 0.2–40 mm gradation exhibited the highest reduction rate, indicating that under a high fine-particle content, the slurry was more likely to form a continuous and dense sealing structure, thereby significantly reducing the connectivity of seepage channels. The 2–40 mm gradation showed the lowest reduction rate, suggesting that although this gradation was more favorable for grout simulant diffusion, some pores still remained insufficiently filled. The 5–40 mm gradation exhibited a relatively high initial permeability coefficient and a moderate reduction magnitude, indicating strong vertical connectivity but a less effective uniform sealing capacity than that of the high fine-particle gradation. Thus, the permeability results were consistent with the analyses of diffusion distance and diffusion rate: increasing the fine-particle content helped improve sealing performance, but may also have reduced diffusion controllability; by contrast, the absence of fine particles facilitated rapid percolation, but was not conducive to the formation of a uniform low-permeability structure.

**Table 5:** Statistical summary of permeability coefficients before and after grouting under different particle-size gradations.

Particle-size gradation	Permeability coefficient before grouting (cm/s)	Permeability coefficient after grouting (cm/s)	Reduction rate of permeability coefficient (%)	P value (before vs after)
0.2 ~ 40 mm	0.61 ± 0.054	0.01 ± 0.002	97.76%	0.0027
2 ~ 40 mm	1.52 ± 0.12	0.66 ± 0.004	56.65%	0.0065
5 ~ 40 mm	1.85 ± 0.09	0.56 ± 0.02	69.83%	0.0012

Note: Data are presented as mean ± standard deviation (SD), with n = 3 independent replicates. The reduction rate of the permeability coefficient was calculated from the permeability coefficients before and after grouting. The P values in the last column were obtained from two-sample t-tests to compare the permeability coefficients before and after grouting for the same particle-size gradation.

## Results and discussion

### Limitations of the study

This study has several limitations. First, although the mean ± standard deviation, one-way ANOVA results, Tukey groupings, and p-values for the permeability coefficients before and after grouting have been reported, the statistical analysis still mainly focuses on variability within repeated tests under the same condition and on pre- and post-grouting changes within the same gradation. Direct comparisons of the same indicators across different conditions remain insufficient. Second, the interpretation of the physical mechanisms is mainly based on experimental observations, such as diffusion distance, peak diffusion rate, variability, and the reduction in permeability coefficient. Although these results support the proposed explanations of pore-throat constriction, local blockage, and low-resistance channel formation, a rigorous porous-medium seepage model or a systematic dimensionless framework has not yet been established. Finally, all experiments were conducted under static water conditions, without considering the effects of dynamic seepage, groundwater chemistry, or further evolution of particle morphology under field conditions.

### Discussion

The results of this study indicate that fine-particle content is a key factor governing the grout simulant diffusion path, diffusion stability, and final sealing performance in coarse-grained materials. Under constant injection pressure and grout simulant viscosity, the differences among the three conditions were mainly caused by variations in pore-throat constriction, pore connectivity, and dominant flow pathways induced by changes in fine-particle content. In Condition 1, the lateral diffusion distance was greater than the vertical diffusion distance, and the lateral standard deviation was also significantly larger, indicating that a high fine-particle content led to local blockage and enhanced medium heterogeneity. As a result, after a short initial downward advance, the slurry was more likely to shift to lateral diversion and surface spreading. In Condition 2, vertical diffusion was significantly enhanced while a certain lateral filling range was still maintained, and good repeatability was observed in the negative x- and positive y-directions. This suggests that an appropriate amount of fine particles not only inhibited the rapid through-connection of large pores, but also avoided excessive blockage, thereby facilitating the formation of a more continuous and balanced diffusion front. In Condition 3, both the final diffusion distance and the peak diffusion rate in the positive y-direction were the highest, and vertical repeatability was also good. This indicates that in the absence of fine-particle constraints, the connectivity of large pores was enhanced, allowing the grout simulant to rapidly infiltrate along low-resistance vertical pathways, although its uniform lateral filling capacity remained relatively limited.

The permeability coefficient results also support this interpretation. The 0.2–40 mm gradation exhibited the highest reduction rate in permeability coefficient after grouting, indicating that a high fine-particle content was more favorable for the formation of a continuous and dense sealing structure. The 2–40 mm gradation showed the lowest reduction rate, suggesting that although this gradation was more favorable for coordinated diffusion and pathway stability, some internal pores were not fully filled. The 5–40 mm gradation exhibited a relatively high initial permeability coefficient and a moderate reduction magnitude, reflecting its strong downward diffusion capacity but limited uniform sealing ability. Therefore, a more reasonable interpretation of the grout simulant diffusion mechanism under different conditions is as follows: by altering the pore structure, fine-particle content governs the competition among pore-throat constriction, local blockage, and the formation of low-resistance pathways, thereby jointly determining grout simulant diffusion direction, repeatability, and final seepage resistance.

In practical engineering, Condition 2 achieved a relatively reasonable balance among diffusion coordination, process stability, and reinforcement uniformity, and may therefore serve as a more suitable reference condition for uniform grouting reinforcement. Condition 1 was more favorable for forming a dense sealing structure, but its diffusion controllability was relatively weak. Condition 3, by contrast, was more suitable for scenarios requiring rapid vertical penetration.

### Conclusion

(1) Fine-particle content significantly affects the diffusion direction, stability, and final sealing performance of grout simulant in coarse-grained materials. Under conditions of high fine-particle content, the grout simulant tends to exhibit enhanced lateral diffusion while vertical penetration is restricted. Under conditions of moderate fine-particle content, the grout simulant exhibits more coordinated diffusion and better repeatability. In the absence of fine particles, the simulant shows the strongest rapid downward diffusion capacity, but its lateral filling capacity is relatively weak.
(2) The mean ± standard deviation, one-way ANOVA results, and Tukey groupings in Tables 2–4 indicate significant differences in the repeatability of the slurry diffusion process under different conditions. In Table 5, the p-values for the comparisons of permeability coefficients before and after grouting were all less than 0.05, indicating that grouting significantly reduced the permeability of each gradation.
(3) Based on the results for diffusion distance, peak diffusion rate, repeatability, and permeability coefficient, a more reasonable interpretation of the grout simulant diffusion mechanism under different conditions is that fine-particle content alters pore connectivity and the degree of pore-throat constriction, thereby regulating the competition among local blockage, diffusion continuity, and the formation of low-resistance channels, and ultimately determining the slurry diffusion pathway and final sealing performance.
(4) From an engineering perspective, Condition 2 achieved a reasonable balance among diffusion coordination, process stability, and reinforcement uniformity, making it more suitable as a reference condition for uniform grouting reinforcement. Condition 1 was more favorable for forming a dense sealing structure, whereas Condition 3 was more suitable for engineering scenarios requiring rapid vertical penetration.

## Supporting information

S1 Dataset Dataset. (XLSX)
